# Cross-sectional imaging for presurgical planning of dialysis circuit vascular access creation in the end stage renal disease patient population

**DOI:** 10.1007/s10554-025-03357-2

**Published:** 2025-03-07

**Authors:** Daniel Raskin, Levester Kirksey, Abraham Levitin, Ali Khalifeh, Jon G. Quatromoni, Sean P. Lyden, Cassandra Kovach, Patrick Ghibes, Amrit Khooblall, Sasan Partovi

**Affiliations:** 1 Interventional Radiology, Cleveland Clinic Main Campus, Cleveland, OH USA; 2 Vascular Surgery, Cleveland Clinic Main Campus, Cleveland, OH USA; 3 Kidney Medicine, Cleveland Clinic Main Campus, Cleveland, OH USA; 4 University Hospital of Tuebingen, Tuebingen, Germany

**Keywords:** Cross-Sectional Imaging, Dialysis Vascular Access, End-Stage Renal Disease (ESRD), Computed Tomography Angiography (CTA), Magnetic Resonance Angiography (MRA)

## Abstract

This systematic review explores the role of cross-sectional imaging modalities—computed tomography angiography (CTA) and magnetic resonance angiography (MRA)—in the preoperative planning of dialysis vascular access for patients with end-stage renal disease (ESRD). A systematic search was conducted using PubMed and Cochrane databases, yielding 45 studies meeting inclusion criteria. These modalities are particularly valuable in cases of complex vascular anatomy, central venous stenosis, and prior surgical interventions. Findings emphasize the advantages of CTA for detailed anatomical mapping and MRA for cases requiring soft-tissue contrast or preservation of renal function. Representative clinical cases illustrate how imaging findings directly influence surgical and endovascular decision-making, optimizing patient outcomes. This manuscript describes the role of cross-sectional imaging for dialysis circuit vascular access interventions including representative clinical examples**.**

## Introduction

End-stage renal disease (ESRD) is a complex medical condition with high incidence and prevalence, necessitating the use of renal replacement therapy (RRT), with the most common type being hemodialysis [1]. Effective hemodialysis requires reliable vascular access, preferably achieved through the creation of an upper extremity arteriovenous fistulas (AVFs) or arteriovenous grafts (AVGs). The maturation rate and longevity of patency of these vascular access points are critical for individual outcomes in the ESRD patient population. Effective access reduces complications and hospitalization rates [2]. Therefore, concise preoperative planning of the dialysis circuit vascular access and assessment are essential for the creation of the best possible arteriovenous fistula or graft in each individual patient [3].

Imaging has a prominent role in the preoperative planning phase. While ultrasound is the mainstay modality for mapping the peripheral upper extremity arterial and venous anatomy, cross-sectional imaging including computed tomography angiography (CTA), and magnetic resonance angiography (MRA) provides detailed insights into vascular anatomy and pathology as problem solving tools, thereby aiding in the selection of optimal sites and type of vascular access in challenging cases [1, 3, 4]. These imaging techniques help identify suitable vessels for dialysis circuit creation and assess their condition, thereby contributing to presurgical planning of AVFs and AVGs [1, 5].

Ultrasound is particularly favored for its high temporal resolution with real-time visualization capabilities, allowing dynamic assessment of vascular structures [3]. It is instrumental in mapping peripheral veins and arteries, ensuring that the selected vessels are adequate for creating a functional dialysis circuit vascular access [1, 6, 7]. On the other hand, CTA and MRA offer comprehensive vascular mapping with high spatial resolution and this is crucial as problem solving tool for complex cases in which detailed anatomical visualization is required, such as those involving central venous stenosis, central arterial pathology or prior surgical interventions [1, 3, 4]. For assessment of dysfunctional access, the guidelines suggest cross-sectional imaging to be particularly valuable when ultrasound findings are inconclusive or when deeper structures, such as central veins, need to be evaluated [6].

This manuscript aims to explore the current state and value of cross-sectional vascular imaging in the context of preoperative planning for dialysis circuit vascular access in the ESRD patient population, highlighting the benefits, limitations, and future directions of CT angiography and MR angiography. Based on our extensive institutional experience with vascular access creation in a large ESRD patient population, we present and discuss representative cases to illustrate key findings.

## Methods

This review was conducted by systematically searching PubMed and Cochrane databases for relevant studies published up to January 2024. Search terms included “vascular access,” “hemodialysis,” “CTA,” and “MRA.” Inclusion criteria were:

- Studies evaluating imaging for dialysis vascular access planning.
- Original research, review articles, and case series with relevant clinical data.
- Publications in English.

Exclusion criteria were:

- Studies without clinical correlation.
- Non-English publications.
- Articles focusing solely on technical imaging methods without patient outcomes.

Out of the initial results, 45 studies met the inclusion criteria and were included in this review.

## Vascular access creation in hemodialysis

AVFs are considered the preferred vascular access option due to their superior long-term patency, lack of synthetic material and lower complication rates compared to other forms of vascular access [8, 9]. The successful creation and maintenance of AVFs are crucial for reducing the time on tunneled dialysis catheters since these catheters are associated with an increased risk of infectious complications leading to worse outcomes in the immunocompromised ESRD patient population [10].

Preoperative imaging plays a pivotal role in the planning and selection of suitable vessels for AVF/AVG creation, thereby improving the chances of long-term success [11]. The National Kidney Foundation’s KDOQI guidelines emphasize prioritizing AVFs due to their durability [6]. The early and accurate assessment of vascular access options through non-invasive imaging techniques, including ultrasound and CTA, is critical for optimizing outcomes and reducing the need for repeated interventions for maturation as well as maintenance after vascular access creation surgery [12, 13]. The longevity and functionality of dialysis circuit significantly impacts the quality of life for hemodialysis patients, as frequent access failures can lead to prolonged hospitalizations and repeated interventions with missed dialysis sessions [1]. Therefore, the selection of the appropriate type of vascular access in each individual patient, combined with effective preoperative planning and ongoing monitoring is critical to achieve the best possible outcome for each individual patients undergoing hemodialysis [14].

## Indication of cross-sectional imaging

Cross-sectional imaging, including CTA and MRA, is especially valuable in cases where central venous or central arterial disease is suspected. These imaging modalities are critical in presurgical planning when complex vascular anatomy, previous surgeries and interventions, or multiple catheter placements are involved. CTA or MRA is preferred over ultrasound in situations in which detailed three-dimensional imaging is necessary to accurately assess the vascular anatomy, particularly when ultrasound findings are inconclusive [15]. A further limitation of US is its inability to image the central venous system. The sternum, clavicle and 1st and 2nd ribs limit visualization of the central chest veins.

Patients with significant calcific atherosclerosis, history of acute aortic syndrome, or prior catheter-related complications are candidates who benefit from cross-sectional imaging, as it provides superior visualization of central vessels and surrounding structures compared to ultrasound. The choice between CTA and MRA is guided by the patient’s specific condition, with CTA as favored cross sectional modality for precise vessel measurement, particularly in central venous stenosis. MRA is preferred for patients with residual renal function to avoid the use of nephrotoxic CT contrast agents, and it is also beneficial for patients with heavy vascular calcifications where blooming artifacts on CTA may obscure critical details [3, 4, 15].

## Cross-sectional imaging modalities

### Computed tomography angiography (CTA)

#### Description

This modality provides an excellent visualization of vascular anatomy and pathology. The generation of thin sliced images allows for multiplanar and three-dimensional reconstruction for the complex cases, especially in patients with prior interventions. [8, 9, 14, 16–19]

## Benefits in presurgical settings

**Fig. 1:**
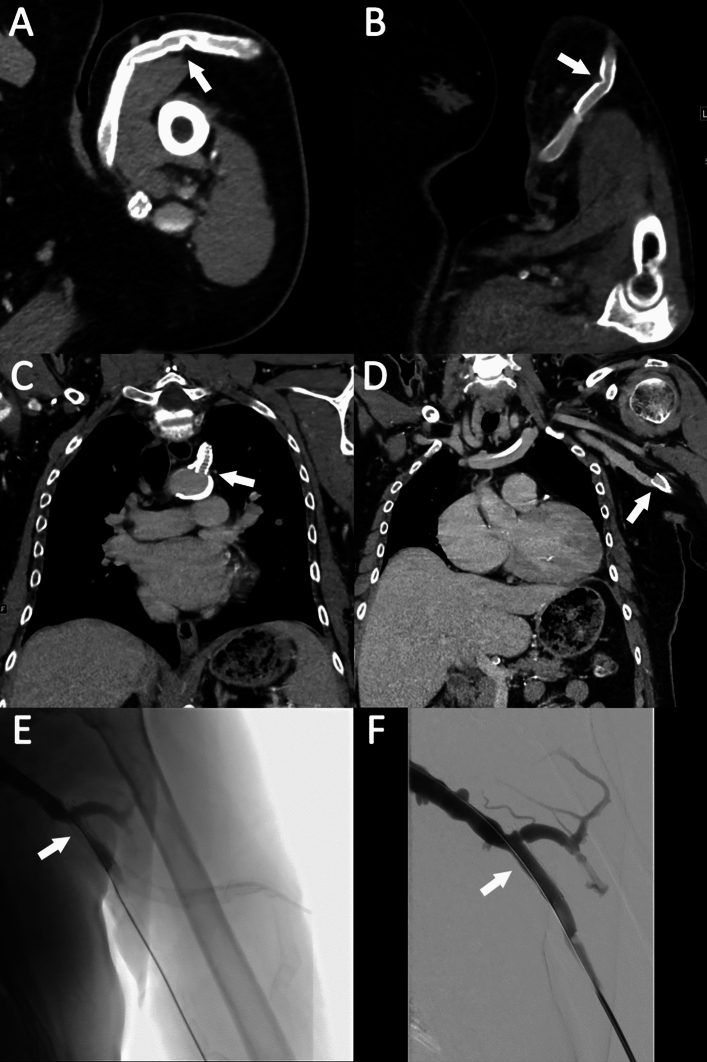
A 80 year-old male with history of multiple left upper extremity dialysis circuit vascular accesses, currently with malfunctioning left upper extremity axillary-axillary loop graft leading to poor flow rates during dialysis and inability to cannulate. CT angiography was performed to assess the current access and evaluate potential new surgical vascular access creation. CT angiography revealed multifocal stenotic disease of the graft within the cannulation zone (arrows) without evidence of complete occlusion (**A** and **B**). No central arterial stenosis was observed, but stenting of the proximal left subclavian artery was noted (arrow in **C**). However, CT angiography revealed a suspected focal high-grade stenosis of the left axillary venous outflow system just proximally to the previously placed stent graft (arrow) (**D**). At this point decision was made to pursue surgical revision with resection of the malfunctioning left upper extremity loop graft. However, given the venous outflow disease involving the left axillary vein, fistulogram was conducted along with endovascular treatment. The subsequent angiography revealed high grade in-stent stenotic disease (**E**). Extension of the stent graft was performed for endovascular reconstruction of the left upper extremity axillary venous outflow system in preparation for surgical revision of the existing left upper extremity axillary-axillary loop graft without evidence of residual stenotic on final digital subtraction angiography images (arrow in **F**)

**Fig. 2:**
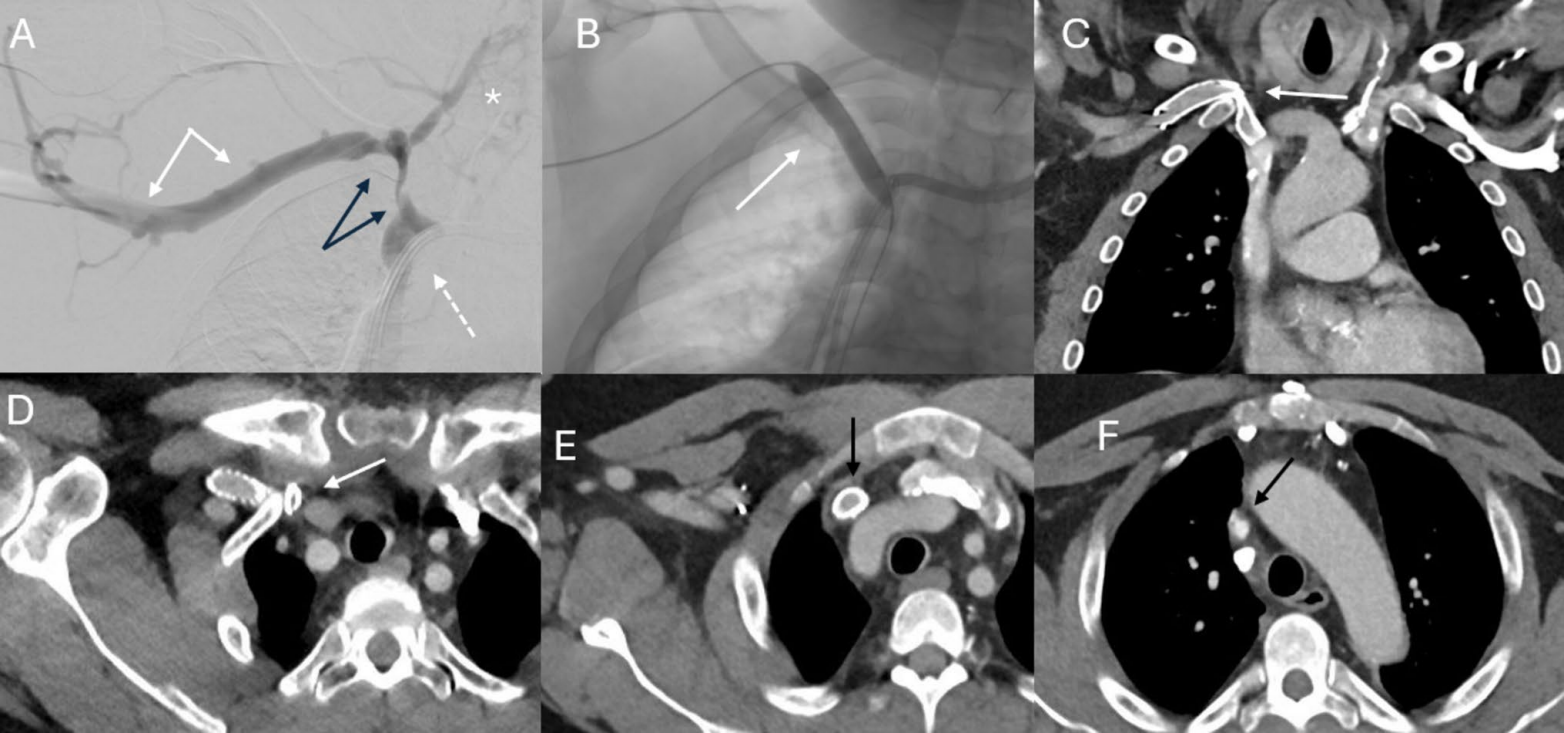
A 52 year-old male with right upper extremity brachiocephalic fistula for hemodialysis who presented with arm swelling and poor flow rates during dialysis. The patient had a history of repeated endovascular interventions. Digital subtraction fistulogram demonstrated patent brachial and axillary veins (white arrows) as well as severe stenotic disease involving the proximal right subclavian vein and right innominate vein (**A**). Collateral venous vasculature visualized and marked with asterisk (**A**). Due to dysfunction of the fistula, a left internal jugular vein tunneled dialysis catheter was placed (white dashed arrow in A). Subsequent balloon angioplasty of the long segment severe stenosis was performed using 12 mm compliant balloon (white arrow in **B**) without stent placement at the time of the procedure. Subsequently, a stent graft was placed at the outside hospital and follow-up CT angiography was performed due to persistently decreased flow rates during hemodialysis in anticipation of surgical vascular access revision. Stent compression with acute kink at the thoracic inlet (white arrows) as seen on coronal reconstruction image (**C**) and axial image (**D**). Caudal portion of the stented right innominate vein is patent (**E**). Mild to moderate stenotic disease of the cranial portion of the superior vena cava is noted (black arrow) on axial slice of the CT angiography study (**F**)

- **Vessel Visualization:** The wide field of view included in CTA is useful when assessing the entire vascular system, including both central and peripheral vessels. This is especially useful in patients with complicated anatomy or a history of prior vascular interventions. CTA can detect luminal stenoses, occlusions, and aneurysms with high spatial resolution and therefore it is the preferred presurgical cross sectional imaging modality in daily clinical practice [4, 15].
- **Measurement Capabilities:** Accurate measurements of vessel diameters is critical for planning AVFs or grafts, as it allows assessment of adequate vessel size, thereby increasing the likelihood of dialysis circuit vascular access maturation [20].
- **Calcification Assessment:** CTA is particularly effective in visualizing the extent of atherosclerotic calcifications of the arterial vasculature. However, heavy calcifications can lead to blooming artifacts on CTA that obscure critical details. In these cases MRA may be considered since vessel evaluation is less affected by atherosclerotic calcifications [21].
- **Challenging Cases:** CTA is especially useful in identifying central venous stenosis or occlusions, which are common in patients who have had multiple catheter placements or a history of previous thoracic surgery or radiation therapy interventions [22].
- **Stent patency:** Stent grafts are associated with minimal artefacts on CTA, thereby enabling evaluation of location, patency and the amount of in-stent thrombosis [18, 23]. In patients with central venous stents, this modality can show the extent of subclavian disease and is best in visualization of subclavian stent patency. Representative examples are shown in Figs. 1 and 2.

## Limitations

The use of nephrotoxic iodinated contrast agents makes it less suitable for patients with CKD with residual renal function, as these agents can accelerate the transition from CKD to ESRD [24–26]. Additionally, in cases with extensive vascular calcifications, the blooming artifacts caused by calcium deposits can obscure smaller arterial vasculature and stenotic disease [27]. Similarly, vascular proximity to osseous structures decreases sensitivity for assessment of central venous stenosis.

## Magnetic resonance angiography (MRA)

### Description

MRA uses gadolinium-based contrast material, compared to iodinated agents used for CTA which are nephrotoxic, making it a more suitable alternative for patients with CKD. Unlike CTA which is dedicated to luminal assessment, MRA provides high-resolution images with soft tissue contrast, allowing for the detailed assessment of both the vascular system and surrounding anatomical structures [15, 23, 28–30]

## Benefits in presurgical settings

**Fig. 3:**
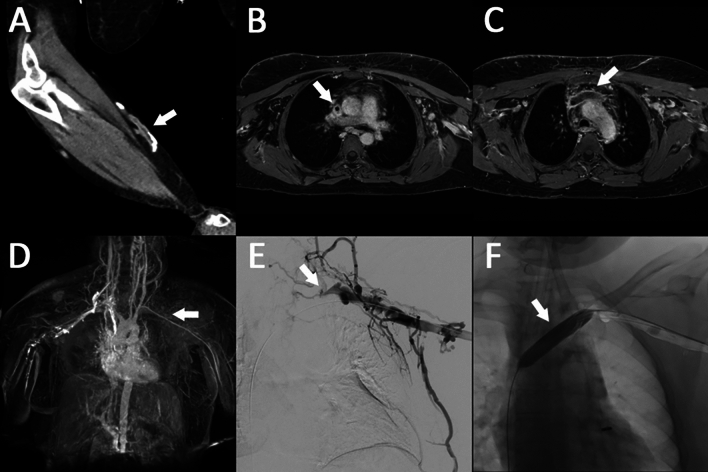
A 72 year-old female with known occlusion of the right upper extremity forearm radiocephalic fistula (arrow in **A**). Left upper extremity swelling occurred after placement of a left tunneled dialysis catheter to continue hemodialysis until surgical creation of a new left upper extremity dialysis circuit vascular access. Non-contrast MR venography was performed for presurgical planning purposes as opposed to CT venography due to renal insufficiency with residual urine production in this patient at the time of the exam. ECG triggered T2 weighted cine sequences with high temporal resolution were acquired for imaging of the central arterial and central venous vasculature. MR venography revealed appropriate positioning of the inserted tunneled dialysis catheter (arrow in **B**). A long segment thrombosis of the severely stenotic left subclavian vein and completely occluded left brachiocephalic vein are visualized (arrows in **C** and **D**). The subsequent angiography confirmed these findings including completely occlusive thrombotic disease of the left brachiocephalic vein and severe stenosis of the left subclavian vein (arrow in **E**). A pharmaco-mechanical thrombectomy with subsequent balloon maceration (arrow) was performed for endovascular reconstruction of the central venous system in anticipation of left upper extremity dialysis circuit vascular access creation (**F**)

- **Soft tissue contrast**: Assessment of soft tissues abutting the vasculature is relevant in detecting perivascular abnormalities such as fibrotic tissue, inflammatory processes or mass lesions, which may impact arterial inflow or outflow. This is particularly beneficial in patients with surgical or radiation therapy history in the chest as well as those with anatomical variations [23].
- **Avoidance of nephrotoxic agents**: One of the primary advantages of MRA is that it utilizes gadolinium-based contrast material which does not exhibit nephrotoxic properties. Moreover, some MR angiography sequences, such as Quiescent-Interval Slice-Selective (QUISS) imaging do not require contrast material and evaluation of the vasculature is diagnostic [30–32]. Representative examples are shown in Fig. 3.
- **Vascular assessment in calcified vessels**: Unlike CTA, MRA is not significantly impacted by blooming artifacts caused by extensive calcifications of the vessel wall. This is relevant in cases where calcification could influence luminal patency assessment, particularly related to assessment of medium and smaller sized vasculature [21].
- **Functional assessment:** In addition to anatomical visualization, MRA protocols offer functional assessment of blood flow dynamics using 4D flow or computational fluid dynamics [33–36].

## Limitations

MR is not as available and specifically MR angiography requires dedicated expertise. Moreover, the image acquisition is lengthier, and this aspect may pose challenges for certain patients, like obese patients, patients with claustrophobia or patients with altered mental status and impaired cognition. Although MRA can provide excellent anatomical and functional data, the resolution is typically lower CTA for detection of small vasculature or subtle stenotic disease. Lastly, the presence of certain implants, such as pacemakers or foreign bodies close to critical structures, must be taken into consideration since some devices are not MR safe and foreign bodies close to critical structures preclude safe performance of MR [37–40].

## Practical input for comparison

**Fig. 4:**
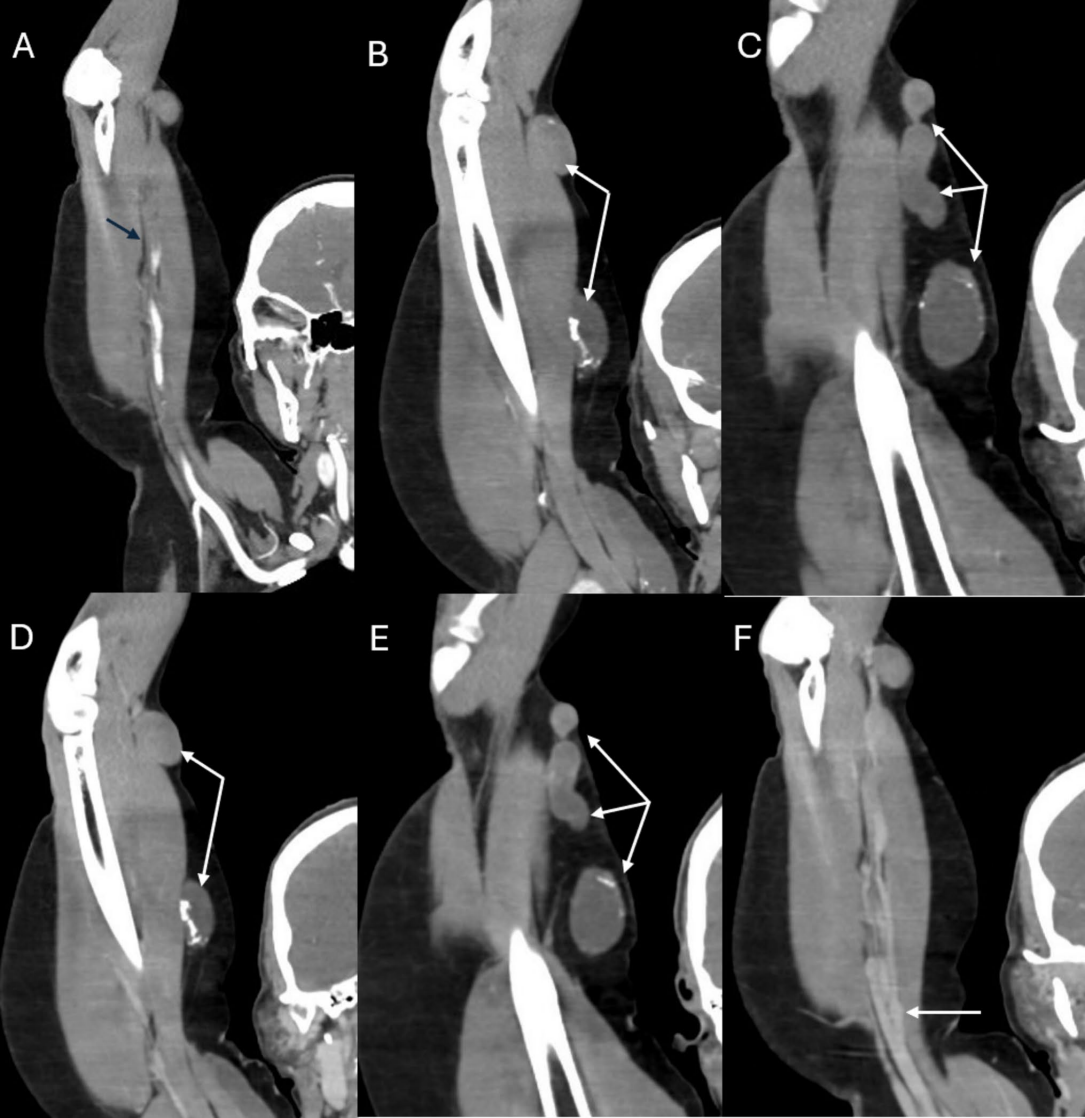
A 61 year-old female with right upper extremity brachiocephalic fistula for hemodialysis who presented with acute upper limb ischemia and therefore computed tomography angiography was performed. Arterial phase coronal reconstruction imaging showed a thrombosed brachial artery (black arrow in **A**). There is lack of opacification of the fistula (white arrows) on arterial phase images (**B** and **C**) as well as venous phase images (**D** and **E**). Patent venous outflow system as seen on venous phase coronal image (white arrow in **F**)

**Fig. 5:**
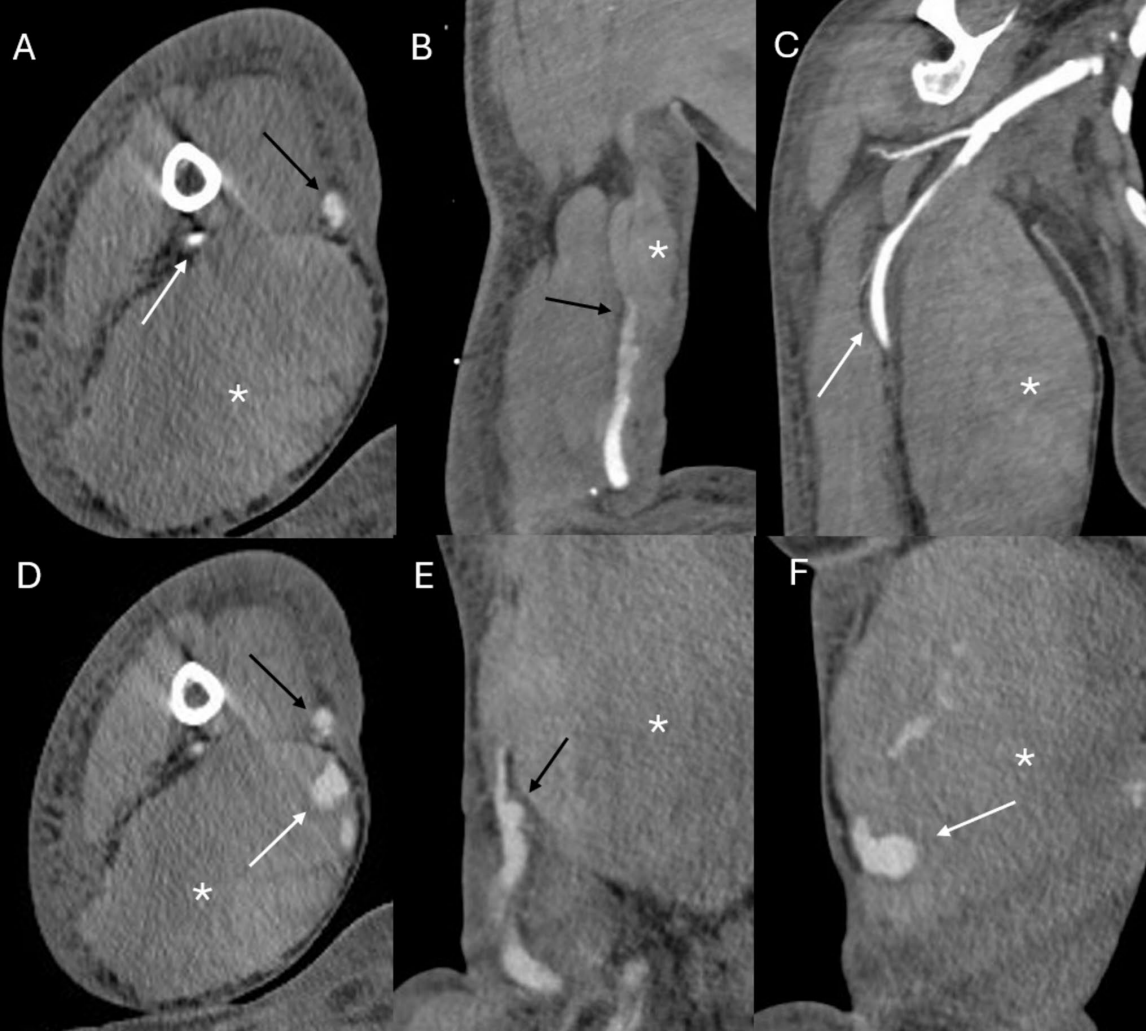
A 61 year-old female with right upper extremity brachiocephalic fistula who presened with expanding hematoma after hemodialysis treatment. Decision was made for surgical revision of the fistula and contrast enhanced computer tomography angiography was obtained in the emergency department for planning purposes. Arterial phase imaging presented a patent brachial artery (white arrow), irregular cephalic vein (black arrow) and a hematoma (white asterisk) on axial image (**A**) and coronal images (**B** and **C**). Venous phase imaging showed contrast material extravasation (white arrows) into the hematoma (asterisk) at the location of the irregularity of the cephalic vein (black arrow) on axial image (**D**) and sagittal images (**E** and **F**). Constellation of findings are consistent with active bleeding requiring urgent surgical revision and fistula excision with subsequent interposition graft placement

Both CTA and MRA are highly effective non-invasive imaging modalities for presurgical planning of dialysis vascular access, though their application is not yet common and depends on the patient’s specific clinical scenario. CTA is often preferred for detailed anatomical visualization, particularly of central vessels, and for precise measurements of vessel diameters. The modalities’ availability and rapid acquisition times make it especially suitable in acute settings, as illustrated in the representative examples shown in Figs. 4 and 5.

MRA, on the other hand, is preferred for more functional assessments of blood flow and hemodynamics. However, its longer image acquisition time and institutional variability in availability may limit its use in certain clinical scenarios.

## Challenges and future directions

Challenges in planning for vascular access creation include the variability in imaging techniques and the potential for inaccurate assessments, particularly when relying on operator dependent ultrasound as the sole modality [8]. This creates the need for standardized protocols and dedicated vascular imaging expertise is required for interpretation of imaging results, especially related to MRA for presurgical dialysis circuit vascular access planning [15]. However, advances in artificial intelligence and machine learning could perhaps assist in image interpretation, leading to more accurate assessments [41–45].

## Conclusion

For specific ESRD patients, cross-sectional imaging is required for preoperative assessment and planning of the dialysis circuit vascular access. Utilization of dedicated CTA or MRA protocols for these patients may potentially increase dialysis circuit maturation, longevity and decrease reintervention rates, thereby leading to superior patient outcomes.

## Data Availability

No datasets were generated or analysed during the current study.
